# Supplementary material for: Impact of the Early Stages of the COVID-19 Pandemic on Coverage of Reproductive, Maternal, and Newborn Health Interventions in Ethiopia: A Natural Experiment
Source: Front Public Health. 2022 Jun 23;10:778413. doi: 10.3389/fpubh.2022.778413 (PMC9260240; doi:10.3389/fpubh.2022.778413)
Supplement: Supplementary file 1 [file Data_Sheet_1.pdf]

Supplementary Material

Supplemental Table 1. Health intervention coverage by cohort at national level

	Births Aug 2019 - Jan 2020			Births May 2020 +		
	**n**	**Proportion**	**95% CI**	**n**	**Proportion**	**95% CI**
Women with 4+ ANC visits	1,550	0.394	[0.346,0.443]	259	0.467	[0.381,0.555]
Among women with any ANC:						
BP check	1,163	0.833	[0.788,0.871]	198	0.842	[0.747,0.905]
Weighed	1,163	0.775	[0.719,0.822]	198	0.799	[0.700,0.871]
Urine test	1,163	0.54	[0.480,0.600]	198	0.578	[0.463,0.685]
Blood test	1,163	0.722	[0.665,0.772]	198	0.756	[0.665,0.829]
Stool test	1,163	0.271	[0.236,0.310]	198	0.304	[0.227,0.394]
Syphilis test	1,163	0.199	[0.157,0.249]	198	0.148	[0.097,0.220]
HIV test	1,163	0.607	[0.545,0.666]	198	0.604	[0.480,0.716]
TT shot	1,163	0.667	[0.610,0.719]	198	0.712	[0.621,0.788]
IFA	1,163	0.756	[0.704,0.802]	198	0.782	[0.683,0.857]
Deworming	1,163	0.168	[0.133,0.210]	198	0.221	[0.151,0.312]
Women that received IFA during pregnancy	1,550	0.627	[0.575,0.675]	259	0.686	[0.580,0.775]
Women that received deworming during pregnancy	1,550	0.137	[0.109,0.171]	259	0.182	[0.122,0.263]
Pregnant women that sought care for: Pregnancy complications	803	0.466	[0.412,0.522]	115	0.653	[0.551,0.742]
Delivery complications	607	0.607	[0.527,0.682]	85	0.754	[0.632,0.846]
Post-delivery complications	491	0.435	[0.361,0.512]	65	0.729	[0.588,0.835]
Women who delivered in a health facility	1,550	0.54	[0.481,0.598]	259	0.555	[0.449,0.656]
Among women delivering in a health facility:						
C-section	956	0.107	[0.086,0.133]	147	0.087	[0.048,0.154]
Blood transfusion	956	0.01	[0.005,0.020]	147	0.016	[0.004,0.061]
Uterotonic use	956	0.735	[0.685,0.780]	147	0.87	[0.759,0.934]
Mother checked after birth	956	0.607	[0.550,0.662]	147	0.648	[0.542,0.741]
Baby resuscitated with ambu bag#	40	0.323	[0.177,0.515]	3	0.889	[0.404,0.989]
Chlorohexidine applied to cord stump	946	0.096	[0.063,0.142]	137	0.072	[0.031,0.159]
Baby weighed at birth	956	0.715	[0.670,0.756]	147	0.769	[0.663,0.850]
Baby checked after birth	967	0.522	[0.465,0.578]	139	0.523	[0.394,0.648]
Skin to skin	967	0.775	[0.731,0.814]	139	0.741	[0.623,0.833]
Delayed bathing	967	0.7	[0.643,0.751]	139	0.795	[0.677,0.877]
Early initiation of breastfeeding	967	0.822	[0.792,0.848]	139	0.803	[0.706,0.874]
Women who received a c-section	1,550	0.058	[0.046,0.073]	259	0.048	[0.027,0.086]
Women who received a uterotonic	1,550	0.411	[0.362,0.461]	259	0.498	[0.391,0.606]
Newborns who had chlorohexidine applied to cord stump	1,533	0.054	[0.035,0.081]	246	0.052	[0.025,0.106]
Newborns receiving skin to skin	1,559	0.461	[0.413,0.510]	249	0.458	[0.355,0.566]
Newborns with delayed bathing	1,559	0.554	[0.497,0.609]	249	0.571	[0.461,0.675]

	Births Aug 2019 - Jan 2020			Births May 2020 +		
	n	Proportion	95% CI	n	Proportion	95% CI
Newborns with early initiation of breastfeeding	1,559	0.793	[0.759,0.824]	249	0.772	[0.701,0.830]
Women who received a postnatal check within first 48 hrs	1,550	0.364	[0.318,0.414]	259	0.424	[0.332,0.520]
Newborns who received a postnatal check within first 48 hrs	1,533	0.316	[0.273,0.362]	246	0.363	[0.268,0.471]
Home visit (or sought care) within first week	1,533	0.13	[0.108,0.156]	246	0.17	[0.110,0.253]
BF counseling during PNC	518	0.442	[0.379,0.508]	89	0.475	[0.331,0.623]
Newborns who received BCG vaccine*	1,559	0.272	[0.231,0.317]	249	0.383	[0.293,0.483]
Newborns who received polio vaccine*	1,559	0.374	[0.330,0.419]	249	0.518	[0.430,0.606]
Newborns exclusively breastfed*	1,517	0.758	[0.722,0.790]	244	0.746	[0.663,0.814]
Sought skilled care for NN illness	546	0.286	[0.229,0.351]	95	0.399	[0.292,0.516]
Women practicing family planning post-delivery*	1,550	0.102	[0.079,0.129]	259	0.164	[0.121,0.220]
Women who intend to practice family planning in next year at time of follow-up interview	1,289	0.749	[0.682,0.805]	203	0.748	[0.668,0.815]

#among children in need of neonatal resuscitation based on maternal report of asphyxia at birth; *at time of follow-up interview

Supplemental Table 2. Health intervention coverage by cohort at national level by urban (Addis and other urban areas) and rural areas

	Rural						Urban						Addis Ababa					
	Births Aug 2019 - Jan 2020			Births May 2020 +			Births Aug 2019 - Jan 2020			Births May 2020 +			Births Aug 2019 - Jan 2020			Births May 2020 +		
	n	Prop	95% CI	n	Prop	95% CI	n	Prop	95% CI	n	Prop	95% CI	n	Prop	95% CI	n	Prop	95% CI
Women with 4+ ANC visits	942	0.317	[0.265,0.373]	185	0.42	[0.326,0.522]	464	0.64	[0.526,0.740]	49	0.652	[0.451,0.811]	144	0.697	[0.469,0.857]	25	0.834	[0.571,0.950]
Among women with any ANC:																		
BP check	666	0.796	[0.738,0.844]	136	0.813	[0.702,0.890]	390	0.938	[0.865,0.972]	40	0.968	[0.753,0.997]	107	0.98	[0.924,0.995]	22	1	-
Weighed	666	0.717	[0.646,0.779]	136	0.767	[0.651,0.853]	390	0.944	[0.882,0.974]	40	0.935	[0.728,0.987]	107	0.961	[0.878,0.988]	22	1	-
Urine test	666	0.481	[0.408,0.555]	136	0.537	[0.405,0.664]	390	0.698	[0.579,0.796]	40	0.743	[0.493,0.896]	107	0.825	[0.664,0.919]	22	0.854	[0.583,0.961]
Blood test	666	0.649	[0.578,0.713]	136	0.715	[0.606,0.803]	390	0.93	[0.856,0.967]	40	0.935	[0.728,0.987]	107	0.991	[0.935,0.999]	22	1	-
Stool test	666	0.235	[0.194,0.282]	136	0.292	[0.204,0.399]	390	0.411	[0.338,0.488]	40	0.357	[0.202,0.550]	107	0.206	[0.102,0.374]	22	0.381	[0.225,0.567]
Syphilis test	666	0.17	[0.123,0.230]	136	0.121	[0.069,0.203]	390	0.282	[0.182,0.411]	40	0.277	[0.113,0.534]	107	0.296	[0.162,0.478]	22	0.268	[0.106,0.531]
HIV test	666	0.513	[0.441,0.586]	136	0.524	[0.384,0.661]	390	0.867	[0.759,0.931]	40	0.967	[0.806,0.995]	107	0.99	[0.928,0.998]	22	1	-
TT shot	666	0.645	[0.574,0.709]	136	0.701	[0.594,0.790]	390	0.716	[0.607,0.805]	40	0.748	[0.559,0.874]	107	0.825	[0.712,0.900]	22	0.82	[0.563,0.941]
IFA	666	0.728	[0.662,0.786]	136	0.754	[0.634,0.844]	390	0.827	[0.748,0.885]	40	0.908	[0.710,0.975]	107	0.921	[0.781,0.974]	22	0.944	[0.645,0.994]
Deworming	666	0.174	[0.131,0.228]	136	0.242	[0.162,0.347]	390	0.165	[0.106,0.247]	40	0.143	[0.038,0.411]	107	0.067	[0.024,0.173]	22	0.043	[0.007,0.235]
Women that received IFA during pregnancy	942	0.575	[0.512,0.635]	185	0.65	[0.526,0.757]	464	0.775	[0.695,0.839]	49	0.832	[0.682,0.920]	144	0.927	[0.821,0.973]	25	0.951	[0.680,0.994]
Women that received dewormer during pregnancy	942	0.136	[0.102,0.178]	185	0.198	[0.129,0.292]	464	0.16	[0.106,0.236]	49	0.124	[0.035,0.354]	144	0.051	[0.0]18,0.13]	25	0.038	[0.006,0.215]
Pregnant women that sought care for: Pregnancy complications	519	0.464	[0.397,0.533]	79	0.661	[0.539,0.764]	227	0.442	[0.371,0.515]	23	0.52	[0.319,0.715]						
Delivery complications	381	0.528	[0.436,0.619]	59	0.717	[0.576,0.826]	167	0.908	[0.805,0.960]	17	0.949	[0.655,0.995]	59	0.949	[0.775,0.990]	9	0.907	[0.477,0.991]
Post-delivery complications	346	0.372	[0.291,0.460]	48	0.705	[0.540,0.830]	112	0.745	[0.593,0.854]	10	0.839	[0.432,0.973]	33	0.841	[0.661,0.935]	7	0.87	[0.467,0.981]
Women who delivered in a health facility	942	0.427	[0.359,0.498]	185	0.486	[0.366,0.607]	464	0.899	[0.830,0.942]	49	0.87	[0.572,0.971]	144	0.994	[0.957,0.999]	25	0.958	[0.715,0.995]
Among women delivering in a health facility:																		
C-section	387	0.074	[0.050,0.108]	80	0.039	[0.010,0.139]	426	0.137	[0.097,0.191]	43	0.201	[0.088,0.394]	143	0.261	[0.185,0.355]	24	0.261	[0.124,0.467]
Blood transfusion	387	0.008	[0.003,0.023]	80	0.022	[0.005,0.087]	426	0.016	[0.006,0.042]	43	0	-	143	0	-	24	0	-
Uterotonic use	387	0.739	[0.660,0.806]	80	0.861	[0.703,0.942]	426	0.752	[0.686,0.808]	43	0.913	[0.776,0.970]	143	0.618	[0.515,0.712]	24	0.827	[0.608,0.937]
Mother checked after birth	387	0.568	[0.492,0.641]	80	0.629	[0.493,0.747]	426	0.64	[0.523,0.742]	43	0.681	[0.477,0.834]	143	0.801	[0.671,0.888]	24	0.759	[0.542,0.893]
Baby resuscitated with ambu bag#	17	0.329	[0.148,0.581]	1	1	-	15	0.282	[0.050,0.746]	1	1	-	8	0.404	[0.140,0.738]	1	0	-
Chlorohexidine applied to cord stump	381	0.104	[0.063,0.167]	72	0.094	[0.038,0.212]	422	0.099	[0.041,0.217]	41	0.01	[0.002,0.050]	143	0.006	[0.001,0.051]	24	0.038	[0.005,0.233]
Baby weighed at birth	387	0.635	[0.573,0.692]	80	0.74	[0.596,0.846]	426	0.834	[0.746,0.895]	43	0.798	[0.651,0.893]	143	0.863	[0.778,0.919]	24	1	-
Baby checked after birth	392	0.496	[0.425,0.567]	74	0.487	[0.322,0.654]	429	0.551	[0.427,0.668]	41	0.573	[0.364,0.759]	146	0.612	[0.463,0.742]	24	0.754	[0.473,0.912]
Skin to skin	392	0.765	[0.697,0.821]	74	0.758	[0.594,0.870]	429	0.798	[0.734,0.849]	41	0.691	[0.506,0.830]	146	0.756	[0.663,0.829]	24	0.718	[0.544,0.845]
Delayed bathing	392	0.688	[0.604,0.761]	74	0.775	[0.617,0.881]	429	0.664	[0.574,0.743]	41	0.797	[0.588,0.916]	146	0.974	[0.933,0.990]	24	1	-
Early initiation of breastfeeding	392	0.822	[0.780,0.858]	74	0.787	[0.661,0.876]	429	0.84	[0.786,0.882]	41	0.82	[0.597,0.933]	146	0.731	[0.644,0.803]	24	0.925	[0.761,0.980]

	Rural						Urban						Addis Ababa					
	Births Aug 2019 - Jan 2020			Births May 2020 +			Births Aug 2019 - Jan 2020			Births May 2020 +			Births Aug 2019 - Jan 2020			Births May 2020 +		
	n	Prop	95% CI	n	Prop	95% CI	n	Prop	95% CI	n	Prop	95% CI	n	Prop	95% CI	n	Prop	95% CI
Women who received a c-section	942	0.032	[0.021,0.047]	185	0.019	[0.005,0.069]	464	0.123	[0.085,0.175]	49	0.174	[0.077,0.350]	144	0.26	[0.183,0.354]	25	0.25	[0.118,0.454]
Women who received a uterotonic	942	0.33	[0.275,0.392]	185	0.432	[0.312,0.562]	464	0.688	[0.618,0.751]	49	0.822	[0.652,0.919]	144	0.615	[0.515,0.706]	25	0.793	[0.572,0.916]
Newborns who had chlorohexidine applied to cord stump	930	0.044	[0.026,0.074]	174	0.06	[0.028,0.126]	459	0.099	[0.042,0.214]	47	0.009	[0.002,0.043]	144	0.006	[0.001,0.051]	25	0.036	[0.005,0.226]
Newborns receiving skin to skin	946	0.373	[0.318,0.433]	177	0.411	[0.293,0.540]	466	0.748	[0.685,0.801]	47	0.677	[0.502,0.813]	147	0.752	[0.663,0.823]	25	0.73	[0.557,0.853]
Newborns with delayed bathing	946	0.509	[0.439,0.578]	177	0.521	[0.395,0.645]	466	0.648	[0.561,0.725]	47	0.763	[0.583,0.882]	147	0.971	[0.933,0.990]	25	1	-
Newborns with early initiation of breastfeeding	946	0.785	[0.740,0.823]	177	0.753	[0.671,0.820]	466	0.838	[0.792,0.876]	47	0.845	[0.649,0.941]	147	0.732	[0.645,0.805]	25	0.928	[0.768,0.981]
Women who received a postnatal check within first 48 hrs	942	0.276	[0.225,0.334]	185	0.379	[0.277,0.492]	464	0.627	[0.519,0.724]	49	0.597	[0.388,0.775]	144	0.816	[0.689,0.899]	25	0.8	[0.581,0.920]
Newborns who received a postnatal check within first 48 hrs	930	0.238	[0.193,0.290]	174	0.321	[0.214,0.450]	459	0.562	[0.451,0.667]	47	0.522	[0.316,0.721]	144	0.618	[0.468,0.748]	25	0.722	[0.455,0.890]
Home visit (or sought care) within first week	930	0.08	[0.059,0.109]	174	0.158	[0.091,0.259]	459	0.267	[0.197,0.351]	47	0.191	[0.090,0.358]	144	0.424	[0.295,0.565]	25	0.361	[0.170,0.610]
BF counseling during PNC	209	0.395	[0.306,0.491]	60	0.458	[0.294,0.631]	220	0.453	[0.350,0.560]	16	0.526	[0.195,0.836]	89	0.753	[0.659,0.828]	13	0.608	[0.286,0.857]
Newborns who received BCG vaccine*	946	0.162	[0.121,0.214]	177	0.337	[0.235,0.456]	466	0.569	[0.452,0.679]	47	0.581	[0.393,0.748]	147	0.946	[0.902,0.971]	25	0.711	[0.409,0.898]
Newborns who received polio vaccine*	946	0.281	[0.234,0.333]	177	0.459	[0.358,0.563]	466	0.626	[0.504,0.733]	47	0.759	[0.547,0.891]	147	0.944	[0.897,0.970]	25	0.967	[0.773,0.996]
Newborns exclusively breastfed*	917	0.763	[0.718,0.803]	174	0.736	[0.639,0.814]	455	0.773	[0.708,0.828]	45	0.84	[0.628,0.942]	145	0.571	[0.469,0.668]	25	0.646	[0.432,0.814]
Sought skilled care for NN illness	352	0.238	[0.174,0.317]	68	0.389	[0.268,0.527]	144	0.435	[0.313,0.565]	16	0.367	[0.151,0.652]	50	0.565	[0.396,0.719]	11	0.664	[0.392,0.859]
Women practicing family planning post-delivery*	942	0.076	[0.052,0.111]	185	0.123	[0.080,0.184]	464	0.174	[0.125,0.238]	49	0.288	[0.155,0.473]	144	0.252	[0.189,0.327]	25	0.652	[0.361,0.861]
Women who intend to practice family planning in next year at time of follow-up interview	817	0.719	[0.637,0.788]	162	0.749	[0.663,0.819]	367	0.847	[0.764,0.905]	32	0.722	[0.389,0.914]	105	0.927	[0.828,0.971]	9	0.893	[0.658,0.973]

#among children in need of neonatal resuscitation based on maternal report of asphyxia at birth; *at time of follow-up interview

Supplemental Table 3. Odds of intervention receipt in COVID-19 impacted cohort (April 2020+ births) versus unaffected reference cohort (Aug 2019 – Feb 2020 births) at national level

	Unadjusted			Adjusted		
	n	OR	95% CI	n	AOR	95% CI
Stillbirths	1264	2.33	[1.04-5.22]	1262	2.58	[1.04-6.43]
Neonatal deaths	2292	1.81	[0.94-3.47]	2289	1.46	[0.80-2.67]
Women with 4+ ANC visits	2289	1.49	[1.14-1.97]	2286	1.72	[1.26-2.35]
Among women with any ANC:						
BP check	1734	1.14	[0.74-1.76]	1734	1.13	[0.72-1.78]
Weighed	1734	0.97	[0.64-1.45]	1734	0.98	[0.62-1.53]
Urine test	1734	1.18	[0.83-1.67]	1734	1.3	[0.91-1.86]
Blood test	1734	1.09	[0.82-1.46]	1734	1.27	[0.90-1.80]
Stool test	1734	1.25	[0.94-1.65]	1734	1.33	[0.98-1.80]
Syphilis test	1734	0.94	[0.65-1.36]	1734	0.95	[0.63-1.42]
HIV test	1734	1.03	[0.72-1.48]	1734	1.2	[0.80-1.80]
TT shot	1734	1.42	[0.98-2.06]	1734	1.38	[0.94-2.03]
IFA	1734	1.12	[0.74-1.69]	1734	1.17	[0.76-1.80]
Deworming	1734	1.22	[0.84-1.78]	1734	1.26	[0.87-1.82]
Women that received IFA during pregnancy	2289	1.24	[0.91-1.67]	2286	1.35	[0.97-1.88]
Women that received dewormer during pregnancy	2289	1.24	[0.86-1.79]	2286	1.28	[0.90-1.84]
Pregnant women that sought care for: Pregnancy complications	1121	1.92	[1.34-2.77]	1121	1.94	[1.34-2.83]
Delivery complications	854	1.62	[1.05-2.50]	854	1.6	[1.01-2.55]
Post-delivery complications	685	3.2	[1.98-5.16]	685	3.08	[1.89-5.03]
Women who delivered in a health facility	2289	1.01	[0.78-1.31]	2286	1.04	[0.75-1.45]
Among women delivering in a health facility:						
C-section	1406	0.83	[0.51-1.36]	1403	0.92	[0.55-1.54]
Blood transfusion	1406	1.7	[0.49-5.88]	1104	1.58	[0.50-5.04]
Uterotonic use	1406	1.53	[0.90-2.59]	1403	1.63	[0.96-2.77]
Mother checked after birth	1406	1.11	[0.81-1.53]	1406	1.19	[0.87-1.62]
Baby resuscitated with ambu bag#	59	1.85	[0.34-10.12]	54	0.35	[0.01-13.66]
Chlorohexidine applied to cord stump	1382	1.21	[0.66-2.22]	1382	1.22	[0.66-2.25]
Baby weighed at birth	1406	1.12	[0.70-1.81]	1403	1.19	[0.73-1.92]
Baby checked after birth	1413	1.15	[0.82-1.61]	1413	1.19	[0.85-1.67]
Skin to skin	1413	0.73	[0.46-1.18]	1410	0.74	[0.47-1.19]
Delayed bathing	1413	1.46	[0.95-2.24]	1410	1.57	[1.00-2.46]
Early initiation of breastfeeding	1413	0.87	[0.60-1.25]	1410	0.93	[0.64-1.36]
Women who received a c-section	2289	0.84	[0.52-1.36]	2274	0.91	[0.54-1.53]
Women who received a uterotonic	2289	1.18	[0.91-1.53]	2286	1.31	[0.99-1.75]
Newborns who had chlorohexidine applied to cord stump	2254	1.24	[0.73-2.13]	2251	1.29	[0.78-2.13]
Newborns receiving skin to skin	2293	0.92	[0.68-1.25]	2290	0.96	[0.68-1.36]
Newborns with delayed bathing	2293	1.13	[0.86-1.49]	2290	1.2	[0.90-1.60]
Newborns with early initiation of breastfeeding	2293	0.87	[0.67-1.11]	2290	0.9	[0.70-1.16]
Women who received a postnatal check within first 48 hrs	2289	1.19	[0.94-1.51]	2286	1.3	[1.00-1.68]

	Unadjusted			Adjusted		
	n	OR	95% CI	n	AOR	95% CI
Newborns who received a postnatal check within first 48 hrs	2254	1.23	[0.94-1.61]	2251	1.32	[0.98-1.77]
Home visit (or sought care) within first week	2254	1.2	[0.80-1.81]	2251	1.33	[0.89-2.01]
BF counseling during PNC	785	1.52	[0.97-2.40]	783	1.49	[0.96-2.32]
Newborns who received BCG vaccine*+	1498	1.12	[0.69-1.83]	1498	1.51	[0.81-2.84]
Newborns who received polio vaccine*+	1498	0.9	[0.57-1.42]	1498	1.01	[0.60-1.67]
Newborns exclusively breastfed*+	1460	0.89	[0.55-1.44]	1460	1.08	[0.66-1.76]
Sought skilled care for NN illness*+	584	1.25	[0.65-2.38]	580	1.24	[0.59-2.61]
Women practicing family planning post-delivery*+	1492	1.21	[0.69-2.13]	1492	1.17	[0.64-2.15]
Women who intend to practice family planning in next year at time of follow-up interview+	1309	0.98	[0.64-1.51]	1309	1.04	[0.63-1.73]

#among children in need of neonatal resuscitation based on maternal report of asphyxia at birth; *at time of follow-up interview; +restricted to follow-up interviews between 5-10 weeks post birth

Supplemental Table 4. Odds of intervention receipt in COVID-19 impacted cohort (April 2020+ births) versus unaffected reference cohort (Aug 2019 – Feb 2020 births) by urban (Addis and other urban areas) and rural areas

	Rural						Urban						Addis					
	Unadjusted			Adjusted			Unadjusted			Adjusted			Unadjusted			Adjusted		
	n	OR	95% CI	n	OR	95% CI	n	OR	95% CI	n	OR	95% CI	n	OR	95% CI	n	OR	95% CI
Stillbirths	815	2	[0.83-4.81]	813	2.05	[0.76-5.52]	338	5.5	[0.52-58.64]	338	6.14	[6.14-6.14]	111	-	-	111	-	-
Neonatal deaths	1408	1.91	[0.93-3.94]	1405	1.53	[0.78-2.99]	663	1.29	[0.23-7.29]	663	1.36	[1.36-1.36]	221	0	-	221	0	-
Women with 4+ ANC visits	1409	1.77	[1.26-2.49]	1406	1.86	[1.29-2.70]	661	1.04	[0.58-1.85]	661	0.99	[0.56-1.77]	219	1.92	[0.62-5.95]	219	2.71	[0.90-8.11]
Among women with any ANC:																		
BP check	1006	1.19	[0.74-1.91]	1006	1.08	[0.67-1.76]	559	1.57	[0.58-4.24]	555	1.94	[0.75-4.98]	130	1	-	84	1	-
Weighed	1006	1.07	[0.68-1.67]	1006	0.98	[0.60-1.59]	559	0.65	[0.18-2.37]	540	0.69	[0.17-2.75]	130	1	-	84	1	-
Urine test	1006	1.2	[0.81-1.80]	1006	1.23	[0.82-1.85]	559	1.62	[0.63-4.15]	544	1.72	[0.71-4.20]	169	1.27	[0.23-7.06]	165	1.85	[0.30-11.29]
Blood test	1006	1.2	[0.86-1.67]	1006	1.27	[0.87-1.84]	559	1.05	[0.45-2.41]	555	1.2	[0.47-3.08]	130	1	-	9	1	-
Stool test	1006	1.35	[0.95-1.92]	1006	1.32	[0.92-1.90]	559	1.01	[0.56-1.85]	555	1.05	[0.60-1.84]	169	2.6	[1.03-6.52]	165	3.55	[1.18-10.68]
Syphilis test	1006	0.96	[0.58-1.58]	1006	0.91	[0.53-1.56]	559	1	[0.53-1.89]	555	1.05	[0.49-2.27]	169	1.32	[0.49-3.59]	165	1.4	[0.46-4.27]
HIV test	1006	1.08	[0.71-1.63]	1006	1.09	[0.70-1.71]	559	3.2	[1.09-9.38]	555	4.84	[1.69-13.90]	169	0.38	[0.02-6.23]	111	0.48	[0.09-2.47]
TT shot	1006	1.5	[0.96-2.35]	1006	1.46	[0.93-2.30]	559	1.21	[0.66-2.23]	559	1	[0.49-2.06]	169	1.18	[0.36-3.86]	167	1.01	[0.29-3.57]
IFA	1006	1.08	[0.67-1.74]	1006	1.07	[0.65-1.74]	559	1.99	[0.73-5.37]	529	1.92	[0.74-4.93]	169	0.8	[0.26-2.47]	144	0.7	[0.19-2.58]
Deworming	1006	1.35	[0.88-2.08]	1006	1.44	[0.94-2.20]	559	0.72	[0.35-1.49]	533	0.7	[0.36-1.39]	169	0.43	[0.04-5.10]	97	0.21	[0.02-2.62]
Women that received IFA during pregnancy	1409	1.26	[0.89-1.78]	1406	1.28	[0.88-1.86]	661	1.75	[0.86-3.55]	657	1.75	[0.86-3.56]	219	0.92	[0.30-2.84]	209	0.99	[0.30-3.22]
Women that received dewormer during pregnancy	1409	1.42	[0.94-2.14]	1406	1.51	[1.01-2.27]	661	0.66	[0.32-1.36]	648	0.64	[0.30-1.36]	219	0.49	[0.04-5.51]	122	0.3	[0.03-2.93]
Pregnant women that sought care for:<br>Pregnancy complications	723	1.92	[1.25-2.95]	723	1.95	[1.24-3.05]	311	1.86	[0.83-4.20]	306	2.16	[0.85-5.48]	87	1.78	[0.50-6.34]	75	2.33	[0.69-7.89]
Delivery complications	534	1.87	[1.17-3.00]	534	1.61	[0.99-2.62]	235	0.85	[0.12-6.03]	233	0.65	[0.10-4.15]	85	0.82	[0.05-13.03]	58	0.68	[0.02-30.29]
Post-delivery complications	477	3.36	[1.94-5.81]	477	2.89	[1.68-4.98]	159	3.06	[0.73-12.79]	142	6.02	[0.92-39.33]	49	1.94	[0.18-20.41]	32	1.57	[0.08-31.55]
Women who delivered in a health facility	1409	1.18	[0.86-1.61]	1406	1.07	[0.76-1.51]	661	0.73	[0.26-2.03]	661	0.74	[0.28-1.94]	219	0.47	[0.06-3.62]	146	0.51	[0.05-5.53]
Among women delivering in a health facility:																		
C-section	583	0.66	[0.23-1.90]	580	0.73	[0.24-2.19]	610	1.23	[0.62-2.43]	598	1.28	[0.60-2.74]	213	0.99	[0.51-1.95]	213	1.07	[0.50-2.27]
Blood transfusion	583	2.1	[0.37-11.99]	583	-	-	610	0.81	[0.06-10.24]	610	-	-	213	-	-	213	-	-
Uterotonic use	583	1.22	[0.60-2.46]	580	1.23	[0.58-2.59]	610	2.74	[1.34-5.61]	608	2.84	[1.35-6.00]	213	3.11	[1.17-8.25]	209	3.36	[1.18-9.56]
Mother checked after birth	583	1.06	[0.71-1.57]	583	1.06	[0.72-1.57]	610	1.6	[0.92-2.79]	608	1.84	[0.92-3.67]	213	1.1	[0.42-2.85]	213	1.09	[0.40-2.96]
Baby resuscitated with ambu bag#	29	-	-	29	-	-	20	-	-	20	-	-	11	-	-	11	-	-
Chlorohexidine applied to cord stump	567	1.34	[0.68-2.65]	567	1.39	[0.70-2.74]	603	0.58	[0.14-2.44]	475	0.47	[0.09-2.50]	212	4.27	[2.84-6.43]	212	-	-
Baby weighed at birth	583	1.43	[0.78-2.64]	580	1.4	[0.75-2.60]	610	0.63	[0.30-1.32]	610	0.6	[0.26-1.35]	213	2.71	[0.47-15.63]	213	3.64	[0.63-21.06]
Baby checked after birth	585	1.06	[0.68-1.64]	585	1.04	[0.66-1.64]	613	1.5	[0.82-2.74]	611	1.66	[0.77-3.57]	215	1.82	[0.63-5.30]	215	2.1	[0.64-6.95]
Skin to skin	585	0.78	[0.40-1.56]	582	0.77	[0.39-1.51]	613	0.61	[0.34-1.12]	611	0.58	[0.31-1.11]	215	0.76	[0.41-1.42]	215	0.8	[0.40-1.58]
Delayed bathing	585	1.38	[0.77-2.47]	582	1.56	[0.82-2.97]	613	1.76	[0.98-3.14]	607	1.83	[1.05-3.18]	215	1.46	[0.22-9.71]	152	2.09	[0.23-19.08]
Early initiation of breastfeeding	585	0.81	[0.52-1.27]	582	0.88	[0.54-1.45]	613	0.81	[0.37-1.78]	591	0.86	[0.38-1.95]	215	2.8	[1.24-6.33]	215	3.44	[1.28-9.25]

	Rural						Urban						Addis					
	Unadjusted			Adjusted			Unadjusted			Adjusted			Unadjusted			Adjusted		
	n	OR	95% CI	n	OR	95% CI	n	OR	95% CI	n	OR	95% CI	n	OR	95% CI	n	OR	95% CI
Women who received a c-section	1409	0.74	[0.26-2.07]	1394	0.69	[0.23-2.04]	661	1.18	[0.60-2.30]	642	1.28	[0.61-2.69]	219	0.96	[0.48-1.94]	219	1.04	[0.48-2.24]
Women who received a uterotonic	1409	1.2	[0.86-1.66]	1406	1.17	[0.83-1.64]	661	1.9	[1.06-3.40]	661	2.09	[1.19-3.65]	219	2.55	[1.01-6.46]	215	2.73	[1.01-7.36]
Newborns who had chlorohexidine applied to cord stump	1383	1.54	[0.87-2.72]	1380	1.46	[0.86-2.50]	653	0.5	[0.12-2.07]	510	0.59	[0.14-2.43]	218	4.16	[2.79-6.19]	218	-	-
Newborns receiving skin to skin	1409	1.06	[0.73-1.53]	1406	1	[0.66-1.51]	663	0.74	[0.41-1.34]	663	0.71	[0.39-1.31]	221	0.77	[0.41-1.45]	221	0.83	[0.42-1.65]
Newborns with delayed bathing	1409	1.11	[0.81-1.53]	1406	1.13	[0.81-1.57]	663	1.73	[0.98-3.04]	663	1.81	[1.05-3.10]	221	1.5	[0.22-10.28]	158	2.09	[0.19-22.84]
Newborns with early initiation of breastfeeding	1409	0.86	[0.64-1.15]	1406	0.88	[0.66-1.17]	663	0.8	[0.39-1.64]	650	0.81	[0.39-1.67]	221	2.29	[1.11-4.74]	221	2.78	[1.12-6.87]
Women who received a postnatal check within first 48 hrs	1409	1.38	[1.04-1.84]	1406	1.33	[0.99-1.78]	661	1.11	[0.59-2.07]	661	1.2	[0.62-2.32]	219	1.09	[0.35-3.44]	219	1.15	[0.37-3.51]
Newborns who received a postnatal check within first 48 hrs	1383	1.43	[1.02-2.01]	1380	1.35	[0.95-1.93]	653	1.09	[0.57-2.06]	653	1.12	[0.54-2.33]	218	1.51	[0.56-4.04]	218	1.74	[0.59-5.15]
Home visit (or sought care) within first week	1383	1.93	[1.15-3.25]	1380	1.88	[1.12-3.15]	653	0.5	[0.22-1.13]	653	0.54	[0.23-1.29]	218	0.74	[0.28-1.90]	218	0.84	[0.33-2.16]
BF counseling during PNC	353	1.74	[1.01-3.00]	351	1.62	[0.94-2.81]	301	1.33	[0.46-3.88]	296	1.19	[0.40-3.50]	131	0.68	[0.23-1.99]	131	0.9	[0.29-2.87]
Newborns who received BCG vaccine*+	927	1.7	[0.90-3.20]	927	2.06	[1.07-3.95]	441	0.67	[0.21-2.17]	441	0.58	[0.16-2.10]	130	0.08	[0.02-0.47]	116	0.05	[0.01-0.37]
Newborns who received polio vaccine*+	927	0.89	[0.49-1.61]	927	0.92	[0.51-1.66]	441	1.76	[0.43-7.11]	424	1.48	[0.38-5.73]	130	1.09	[0.11-10.74]	130	-	-
Newborns exclusively breastfed*+	900	0.78	[0.46-1.32]	900	0.89	[0.52-1.52]	432	2.08	[0.20-21.16]	429	2.26	[0.24-21.37]	128	2	[0.53-7.50]	126	2.95	[0.60-14.55]
Sought skilled care for NN illness+	385	1.27	[0.57-2.83]	381	1.09	[0.43-2.75]	148	1.05	[0.19-5.86]	143	0.89	[0.22-3.61]	51	2.65	[0.53-13.21]	46	4.23	[0.54-33.14]
Women practicing family planning post-delivery*+	928	1.11	[0.51-2.42]	829	0.84	[0.37-1.91]	437	1.16	[0.27-5.03]	434	0.97	[0.19-5.00]	127	4.14	[1.11-15.47]	125	4.02	[1.02-15.85]
Women who intend to practice family planning in next year at time of follow-up interview+	854	1.11	[0.69-1.78]	854	1.12	[0.64-1.95]	362	0.56	[0.15-2.09]	359	0.48	[0.13-1.84]	93	0.71	[0.09-5.35]	36	0.65	[0.16-2.70]

#among children in need of neonatal resuscitation based on maternal report of asphyxia at birth; *at time of follow-up interview; +restricted to follow-up interviews between 5-10 weeks post birth

Supplemental Figure 1. Time between birth and follow-up interview by date of birth

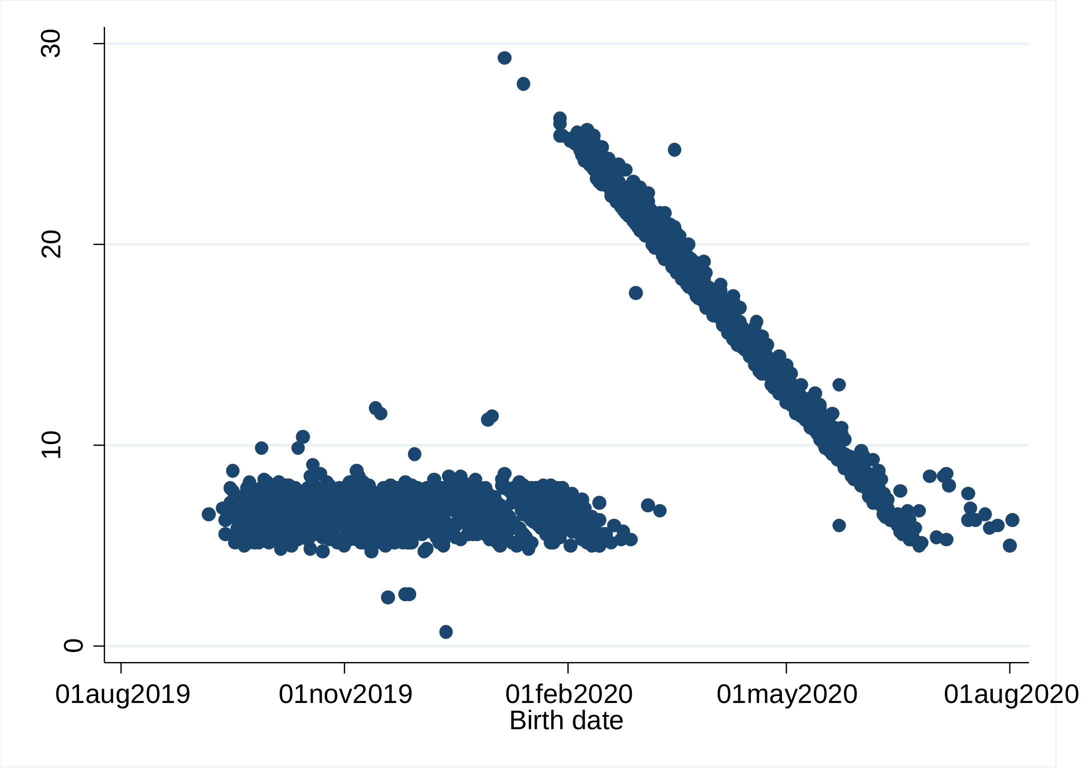

Supplemental Figure 2. Number of births per month included in analysis

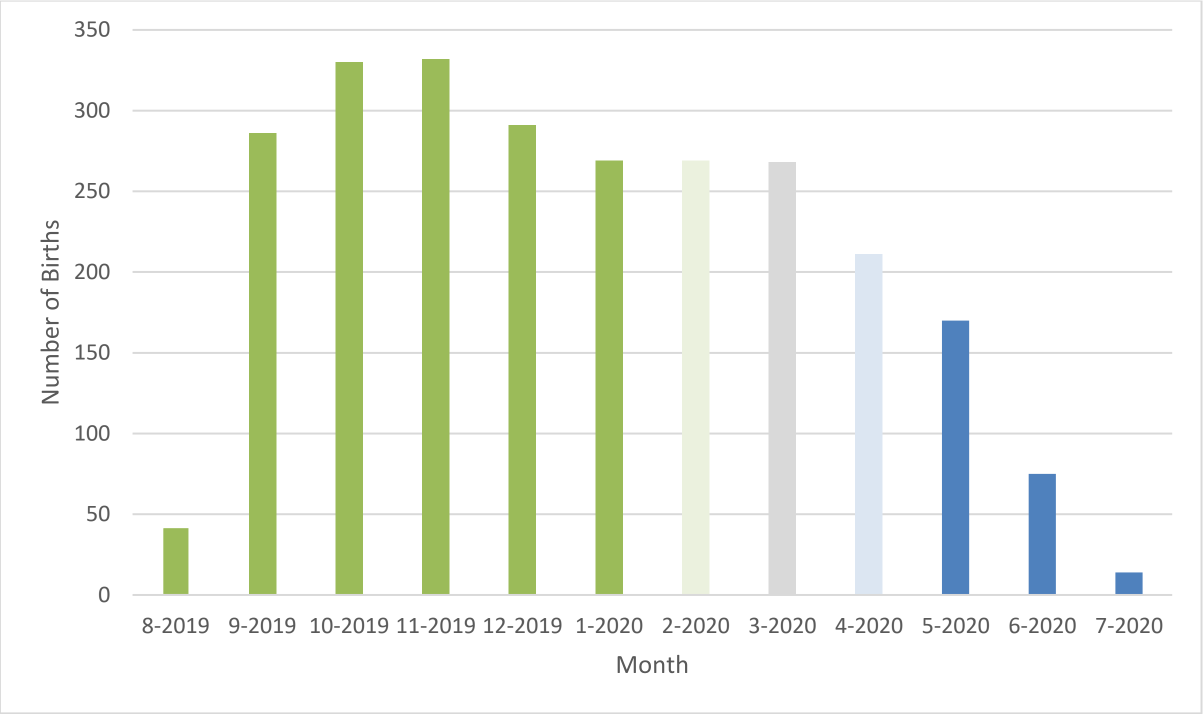

COVID-19 unaffected reference cohort in green. COVID-19 affected cohort in blue. Lighter shading indicates births only included in sensitivity analysis. March 2020 births (grey) excluded from all analyses.
